# Parasite infection and host personality: *Glugea*-infected three-spined sticklebacks are more social

**DOI:** 10.1007/s00265-018-2586-3

**Published:** 2018-10-06

**Authors:** Irina Petkova, Robin N. Abbey-Lee, Hanne Løvlie

**Affiliations:** 1 Department of Physics, Chemistry and Biology, IFM Biology, Linköping University, SE-581 83 Linköping, Sweden; 2 School of Biological Sciences, Centre for Ecology, Evolution and Behaviour, Royal Holloway University of London, Egham, TW20 0EX UK

**Keywords:** Animal personality, *Glugea anomala*, Exploration, Fish, Parasite infection, Sociability

## Abstract

**Abstract:**

The existence of animal personality is now well-documented, although the causes and consequences of this phenomenon are still largely unclear. Parasite infection can have pervasive effects on hosts, including altering host behaviour, and may thus contribute to differences in host personality. We investigated the relationship between the three-spined stickleback and its common parasite *Glugea anomala*, with focus on differences in host personality. Naturally infected and uninfected individuals were assayed for the five personality traits activity, exploration, boldness, sociability, and aggression. If infected fish behaved differently from uninfected, to benefit this parasite with horizontal transmission, we predicted behaviour increasing interactions with other sticklebacks to increase. Infection status explained differences in host personality. Specifically, *Glugea*-infected individuals were more social than uninfected fish. This confirms a link between parasite infection and host behaviour, and a relationship which may improve the horizontal transmission of *Glugea*. However, future studies need to establish the consequences of this for the parasite, and the causality of the parasite-host personality relationship.

**Significance statement:**

Parasite infection that alters host behaviour could be a possible avenue of research into the causes of animal personality. We studied the link between infection and personality using the three-spined stickleback and its parasite *Glugea anomala*. We predicted that infected individuals would be more prone to interact with other sticklebacks, since this would improve transmission of this parasite. The personality of uninfected and naturally infected fish was measured and we observed that *Glugea-*infected sticklebacks were more social. Our results confirm a link between parasitism and variation in host personality.

**Electronic supplementary material:**

The online version of this article (10.1007/s00265-018-2586-3) contains supplementary material, which is available to authorized users.

## Introduction

Animal personality (i.e. consistent among individual differences in behaviour, Dall et al. 2004) has been observed in numerous species across a wide range of taxa (Gosling 2001; Réale et al. 2007; Carere and Maestripieri 2013). Animal personality can influence fitness, thus have ecological and evolutionary consequences (Dall et al. 2004; Smith and Blumstein 2008; Carere and Maestripieri 2013). The observations of consistent behavioural responses oppose the traditional view that behaviour is adaptively adjusted to each situation (Dall et al. 2004; Stamps 2007; Carere and Maestripieri 2013). As a result, with the aim to understand why animals have personality, investigation of the causes and consequences of personality has become an important topic in animal research (Carere and Maestripieri 2013).

Parasitism is a relationship where the parasite benefits at the expense of the host (Zelmer 1998). This is a widespread strategy among living organisms, and consequently, most individuals of all species are exposed to parasite infection (Poulin 2014). Further, parasite infection can cause subtle, to more dramatic, changes to hosts (e.g. reviewed by Adamo 2002; Moore 2002). Subtle changes can be alterations in the frequency at which a behaviour is performed, such as feeding rate, activity (e.g. Christe et al. 1996), or in growth rate (e.g. Stauffer et al. 2017). More dramatic changes include for example when toxoplasmosis (*Toxoplasma gondii*)*-*infected rats become attracted to, rather than repelled by, cat odour (Berdoy et al. 2000; Vyas et al. 2007), or when chimpanzees (*Pan troglodytes trodlodytes*) infected with the same parasite lose their innate aversion to urine of their only natural predator, leopards (*Panthera pardus*, Poirotte et al. 2016). Some examples of host behavioural changes is when fungi-infected ants abandon their colony and position themselves at the top of a grass, thus becoming exposed to being eaten by grazing animals, which are the final hosts (De Bekker et al. 2014), insects infected with hairworms that suicidally jump into water (Ponton et al. 2011), or when the worm *Schistosoma mansoni* enhances positive phototropism in infected freshwater snails (*Biomphalaria glabrata*), increasing their vulnerability to predation (Maeda et al. 2018). These changes can be pathological consequences of infection with no adaptive value to the parasite (Minchella et al. 1985; Moore 2002), be caused by the host to reduce the costs of infection (Poulin 1995), or be an active alteration of host behaviour by the parasite to facilitate transmission (Moore 2002). Such behavioural effects of parasites can thus be categorised according to whether these effects seem to benefit the parasite (parasite ‘manipulation’), benefit the host, or be side effects of infection (parasitic ‘constraints’, Kavaliers et al. 1999, see also Thomas et al. 2005 for review).

Both subtle and conspicuous differences in behaviour between infected and uninfected individuals can potentially explain variation in personality between these individuals. For example, a shift in growth rate can theoretically set individuals off on different life-history trajectories, which are suggested to underlie personality differences (Stamps 2007). Similarly, changes in metabolism, which can be observed in infected individuals (Pasternak et al. 1995; Barber and Dingemanse 2010), are also predicted to underlie differences in personality (Barber and Dingemanse 2010; Réale et al. 2010). Parasite infection can also alter host behaviour more directly, for example, by increasing risk-taking and activity in infected individuals (Barber et al. 2004; Talarico et al. 2017; Poirotte et al. 2016; Maeda et al. 2018), or by causing parasite-induced spatial segregation of individuals in a population (Ponton et al. 2005). Parasite infection typically is a relatively stable ‘state’ (sensu Dall et al. 2004; Barber and Dingemanse 2010), in other words, changes brought on by infection are likely to persist at least over some time (Barber and Dingemanse 2010; Adamo 2013). Therefore, parasitism offers a potential explanation for why individuals in a population behave differently and why these behavioural differences are consistent among individuals (Dall et al. 2004; Barber and Dingemanse 2010; Coats et al. 2010; Poulin 2013).

Parasitism is observed to co-vary with a range of host behaviour that can describe variation in personality: in humans, *T. gondii* infection is related to personality differences (Flegr 2007; but see Worth et al. 2013); in rats, Seoul virus increases aggression (Klein et al. 2004); in Siberian chipmunks (*Tamias sibiricus*), tick load correlated indirectly with exploration and activity (Boyer et al. 2010); proactive feral cats were more likely to be infected with feline immunodeficiency virus than their conspecifics (Natoli et al. 2005); ectoparasitic sea lice (*Lepeophtheirus salmonis*) infection reduced locomotor activity in Atlantic salmon (Øverli et al. 2014); in male rock lizards (*Iberolacerta cyreni*), blood parasite load covaried with risk-taking (Horváth et al. 2016); and in the yellow wagtail (*Motacilla flava*), mixed hæmosporidian infections were associated with higher fearfulness (Marinov et al. 2017). A common framework to describe personality variation is along the five gradients of activity, exploration, boldness, sociability, and aggression (Réale et al. 2007). It is currently unknown how each of these personality traits is affected by parasite infection, but if they are altered by infection, it is likely to be in a parasite-specific manner (due to the different requisites for parasite transmission). Improved insight into how specific personality gradients are affected by parasite infection could therefore indicate the mechanism underlying specific host changes (e.g. overall reduction in activity vs. increased interaction with conspecifics, or exposure to predators).

To shed light on the relationship between host personality and parasite infection, we used the three-spined stickleback (*Gasterosteus aculeatus*). The stickleback is an important model in ecology (Barber 2013), physiology (Kitano et al. 2011), and animal personality (e.g. Dingemanse et al. 2007). Additionally, the relationship between sticklebacks and parasites, mainly the cestode parasite *Schistocephalus solidus*, has been well studied (e.g. Giles 1983; Øverli et al. 2001; Barber and Scharsack 2010). *S. solidus* has a complex life cycle, where sticklebacks are one of two intermediate hosts, and avian predators are the final host needed for parasite reproduction. In this system, infected sticklebacks display reduced antipredator response and increased risk-taking (Giles 1983; Milinski 1985; Barber et al. 2004), a change thought to facilitate parasite transfer. This demonstrates that parasite infection can influence stickleback behaviour of relevance to personality research.

We focused on a common parasite of sticklebacks with a horizontal transmission, *Glugea anomala*, also known to alter the appearance of its host (Milinski 1985; Ward et al. 2005). *G. anomala* is a spore-forming unicellular parasite belonging to the phylum Microsporidia. Microsporids have a wide range of animal hosts (from insects to fish to mammals) and are closely related to fungi, but lack mitochondria and motile structures and have very small genomes (Pombert et al. 2013; Weiss and Becnel 2014). Infection by *G. anomala* occurs when fish ingest either free-floating spores or infected aquatic invertebrates (Weissenberg 1968). These spores germinate and produce tumours (or ‘xenomas’), containing hypertrophied host cells and cells from the parasite (Canning 1977). The life cycle of the parasite is completed when tumours are ruptured by physical trauma and spores that are released into the water encounter another susceptible host (Canning 1977). Thus, it is critical for the transmission of the parasite to be in range of other susceptible hosts at the time of tumour rupture. Previous work showed that sticklebacks infected with *G. anomala* had increased shoaling behaviour (Ward et al. 2005). We here expand on this work by holistically comparing how all five animal personality gradients differ between sticklebacks that are naturally infected by *G. anomala*, and those that are uninfected. If the change in host behaviour is under parasite control, because of the horizontal transmission of *G. anomala*, we predict that infection should preferentially influence behaviours related to interactions with conspecifics (i.e. sociability and aggression) rather than risk-taking or activity-related behaviour.

## Materials and methods

### Study population

Three-spined sticklebacks around Sweden have a 2-year life cycle. We used young (≤ 1 year), wild-caught three-spined sticklebacks collected near Oxelösund, Sweden (58° 40′ N 17° 07′ E) using sink nets, on two occasions (2015-09-01 and 2015-11-11). The fish were caught from three locations located 1–3 km from each other (i.e. with a shared gene pool, Makinen et al. 2006). The fish were transported to the lab at Linköping University and housed in several 27-L (ca 40 × 27 × 27 cm), filtered, plastic aquaria with gravel substrate and plastic plants for enrichment. Aquaria were covered on 4 sides and approximately 50% on top, minimising visual disturbance and providing a sheltered area. Salinity was 0.05‰ and lights were kept on a 16:8 light: dark cycle. Fish were fed on a daily basis with defrosted red bloodworms. Water quality was regularly tested, and tanks received water changes bi-weekly, but more often if necessary. Salinity, temperature, and day length in this experiment were all within the natural range for sticklebacks in Sweden.

Sixty-two fish were used (*n*_population1_ = 22; *n*_population2_ = 20; *n*_population3_ = 20). Within populations, fish were distributed among the aquaria in a way that made visual identification possible (based on size differences, infection status, and/or elastomer tagging). In population 1, all fish were uninfected; in populations 2 and 3, about half of fish were naturally infected by *G. anomala* (8 and 10 infected fish respectively). All fish were initially visually inspected and infection status later confirmed by dissection (see below). Behavioural observations took place between 2015-10-18 and 2016-02-04 when the fish had been in captivity ˃ 6 weeks. Exposure to behavioural assays took place before the daily feeding. One focal animal at a time was observed first in a novel arena followed by a mirror test (see details below). This process was called ‘Trial 1’. When the *Glugea* infection was not visible, data were recorded blind with regard to infection status. Tested fish were removed from their residential tank in a randomised, rotational pattern to minimise the effect of disturbing the same tank repeatedly over a short period of time. After waiting for at least one day, the process was repeated on the same individual, using a different novel environment (‘Trial 2’).

At the end of the experiment, fish were dissected, and fish from populations 2 and 3 (i.e. the populations with infected fish) were measured for length (with 0.1 cm accuracy) and weight (with 0.00001 g accuracy), and parasite status and parasite load. Dissections were done by cutting open each fish and removing internal organs to inspect for *Glugea* cysts to confirm parasite status (yes, no), and measure parasite load (with 0.00001 g accuracy). It was confirmed that no fish had reached sexual maturity during the study.

### Behavioural assays

All behaviour was monitored by direct observation by the same observer, from a distance of ≥ 2 m to avoid direct disturbance of the fish.

#### Novel arena test

A 27-L novel arena tank in trial 1 contained fine white sand, three small piles of stones, and the aquarium was divided into 12 imaginary areas by the use of subtle marks on the outside of the aquaria (two rows of three equal-sized areas in the lower, and similarly six areas in the upper part of the aquaria). To maintain the novelty of the environment, the tank used in trial 2 contained coarse brown sand and the configuration of stones was altered. To measure activity, we recorded whether the fish was active (i.e. swimming) or still, with instantaneous sampling every 20 s over the 15-min duration of the test. To measure exploration, the latency to make the first move and the time it took to explore all 12 areas of the tank were recorded. To measure boldness, the time it took the individual to first explore the upper half of the tank was recorded. All times are recorded in seconds.

#### Mirror test

After the novel arena test, fish were exposed to a 10-min mirror test to measure aggression and sociability towards a same-size conspecific (Scherer et al. 2016; Abbey-Lee et al. 2018). A mirror was placed in the tank to contain the fish in a 1/3 of the tank farthest from the sheltered area. This was done to ensure that a lack of interactions with the mirror image was not due to the fish having not seen it. Latency to approach the mirror was recorded. To measure sociability, time spent in close vicinity (1 body length distance) of the mirror was recorded. To measure aggression, the number of attacks launched at the mirror-image was counted. Our measure of sociability and aggression were differentiated by aggression being when a fish showed physical contact with the mirror with its mouth (it was trying to quite vigorously ‘bite’ the mirror image of a fish), while when for our measure of sociability, it did not show this behaviour.

### Statistical analyses

All statistical analyses were performed with R version 3.3.1 (R Core Team 2016). We applied linear mixed-effects models to analyse our data (Table S1, Supplementary Information), for which we used the ‘lmer’ function (package lme4; Bates et al. 2015). Additionally, we used the ‘sim’ function (package arm; Gelman and Su 2016) to simulate the posterior distribution of the model parameters, and values were extracted based on 2000 simulations (Gelman and Hill 2007). The statistical significance of fixed effects and interactions were assessed based on the 95% credible intervals (CI) around the mean (β). We used visual assessment of the residuals to evaluate model fit.

To investigate if parasite infection was correlated with physical or behavioural aspects of the fish, we used linear models (package lme4; Bates et al. 2015). To explore the relationship between parasite infection and personality, for all behavioural variables, infection status (0 for uninfected/1 for infected) and trial number (1/2) were added as fixed effects, and population (1/2/3), residential tank (1–18), and fish identity were included as random effects.

We determined if parasite weight or parasite load (weight of parasite relative to fish weight) were predicted by fish weight or length. We next determined if fish length, weight, or parasite weight or load predicted their behaviour. For these, we ran independent models for each behaviour as a response variable, and either fish weight, fish length, parasite weight, or parasite load as a fixed effect.

#### Data availability

Data is provided as Supplementary material (Table S1).

## Results

Infected and uninfected fish did not differ in weight (F_1,42_ = 2.03, *p* = 0.16) or length (F_1,42_ = 3.79, *p* = 0.06). Fish size and weight were overall unrelated to variation in measured behaviour (*p* ≥ 0.1); other than that, heavier fish attacked the mirror less (F_1,42_ = 6.26, *p* = 0.02) and spent less time near mirror (F_1,42_ = 10.01, *p* = 0.003), and longer fish spent less time near it (F_1,42_ = 7.72, *p* = 0.008). Neither parasite weight nor parasite load correlated with any measured behaviours (F_1,42_ = 1.90, *p* > 0.17).

Behaviours measured in our personality assays had moderate to high repeatability (*R* = 0.25–0.68, *p* ˂ 0.05) except for latency to explore all squares, latency to approach the mirror, and the number of attacks (*R* < 0.20, *p* ≥ 0.1).

**Fig. 1:**
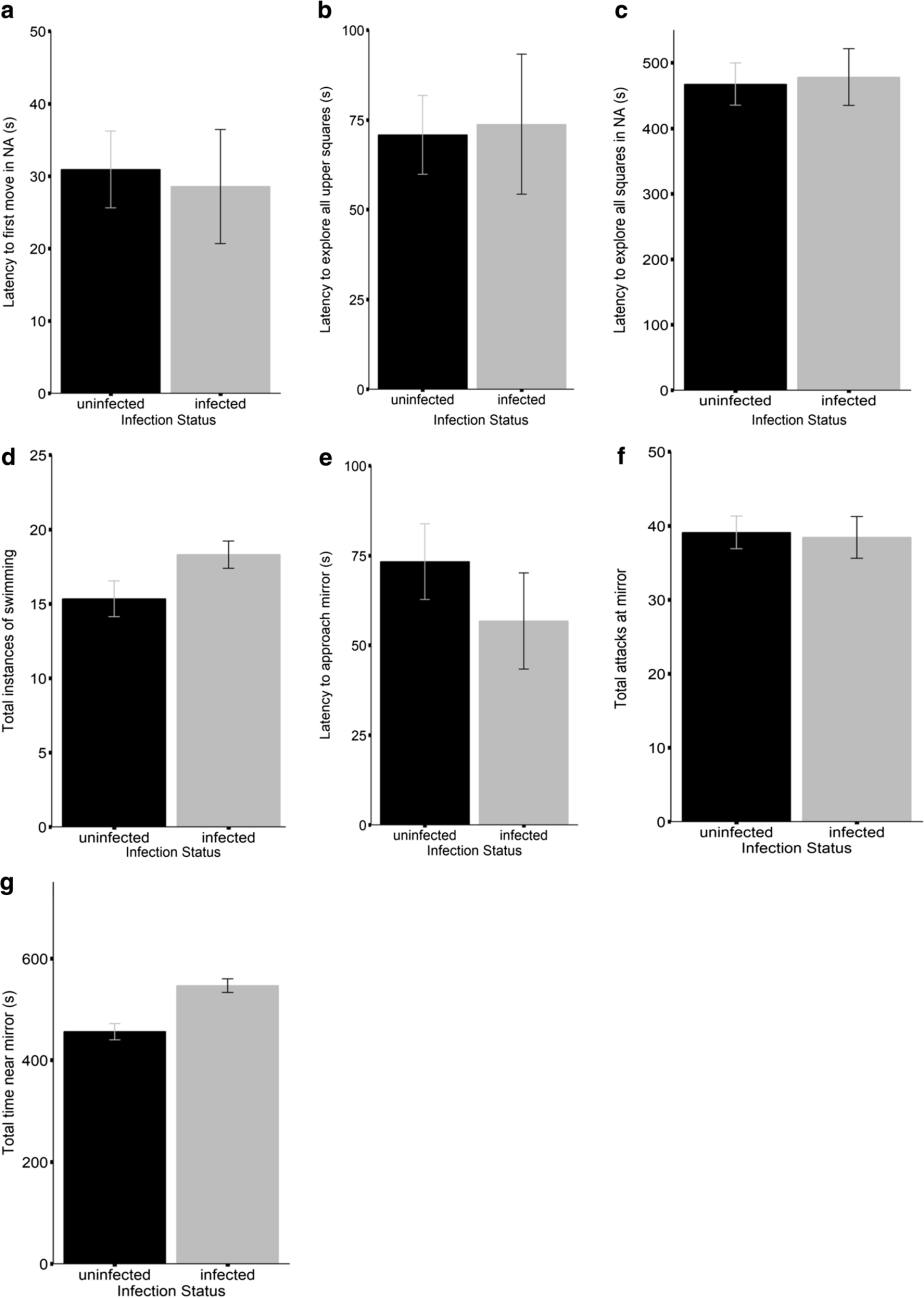
The influence of *Glugea anomala* infection on stickleback behaviour in personality assays. Means and standard errors for each infection status category (black = uninfected, grey = infected) of behaviours assayed in a novel arena: **a** initial response (Latency to Move), **b** latency to explore all upper squares (Latency to Upper Squares), **c** latency to explore all squares (Latency to All Squares), and **d** total time active (Total Instances of Swimming); and when exposed to a mirror: **e** latency to first approach mirror (Latency to Approach Mirror), **f** the number of times the fish poked its reflection (Number Attacks at Mirror), and **g** total time spent in the vicinity of the mirror (Total Time near Mirror)

Fish infected by *G. anomala* were significantly more sociable and somewhat more active and bold than their uninfected conspecifics (Table 1, Fig. 1). In the novel arena, infected fish tended to move sooner and be more active, compared to uninfected fish. Infection status did not explain variation in other behaviour measured in this test. In the mirror test, infected fish tended to approach the mirror faster, while infected fish were more sociable and spent more time in vicinity of the mirror, compared to uninfected fish. The total number of attacks directed at the mirror did not differ between uninfected and infected individuals (Table 1, Fig. 1).

**Table 1:** The influence of *Glugea anomala* infection on stickleback behaviour

	Latency to move	Latency to upper squares	Latency to all squares	Total instances of swimming	Latency to approach mirror	Number attacks at mirror	Total time near mirror
Fixed effects	β (95% CI)	β (95% CI)	β (95% CI)	β (95% CI)	β (95% CI)	β (95% CI)	β (95% CI)
Intercept	2.98 (2.39, 3.60)	3.01 (2.43, 3.56)	444.9 (362.4, 530.0)	19.85 (13.88, 26.14)	3.13 (2.70, 3.57)	36.27 (29.84, 42.88)	439.5 (392.4, 486.6)
Parasitized	*−* 0.46 (− 0.95, 0.05)	0.36 (− 0.70, 1.46)	1.27 (−110.81, 117.30)	2.07 (− 0.48, 4.75)	− 0.48 (− 1.14, 0.15)	0.17 (− 8.14, 8.32)	*93.36* (*37.21*, *152.3*)
Trial 2	− 0.20 (− 0.60, 0.19)	**−** *0.04* (**−** *0.07*, *0.00*)	*87.2* (*9.54*, *163.5*)	− 1.15 (− 2.48, 0.20)	0.53 (− 0.02, 1.13)	4.47 (− 1.67, 10.83)	18.19 (− 22.86, 57.14)
Random effects	σ^2^ (95% CI)	σ^2^ (95% CI)	σ^2^ (95% CI)	σ^2^ (95% CI)	σ^2^ (95% CI)	σ^2^ (95% CI)	σ^2^ (95% CI)
Population	0.10 (0.00, 0.50)	0.00 (0.00, 0.00)	514.9 (19.21, 2589.5)	24.10 (1.99, 124.02)	0.00 (0.00, 0.00)	5.74 (0.19, 31.04)	279.67 (10.12, 1422.23)
Fish ID	0.00 (0.00, 0.00)	4.27 (3.44, 5.45)	0.00 (0.00, 0.00)	11.78 (8.50, 16.43)	0.00 (0.00, 0.00)	42.39 (28.77, 62.53)	5588.0 (3947.4, 7817.1)
Tank ID	0.59 (0.29, 1.12)	0.01 (0.00, 0.02)	2633.8 (1145.3, 5587.3)	0.93 (0.38, 1.85)	0.00 (0.00, 0.00)	22.76 (9.90, 44.56)	2185.6 (986.3, 4280.9)

Estimated effect sizes and 95% credibility intervals (CI) around the mean of predictors of the measured behaviours. In a novel arena: initial response (Latency to move), latency to explore all upper squares (Latency to upper squares), latency to explore all squares (Latency to all squares), and the amount of time active (Total instances of swimming). Exposed to a mirror: latency to first approach mirror (Latency to approach mirror), number of times the fish poked its reflection (Number attacks at mirror) and total time spent in the vicinity of the mirror (Total time near mirror)

Italicized = 95% credible interval does not overlap 0

## Discussion

We have here shown that in wild-caught sticklebacks, behaviour measured in personality assays differed between individuals infected with *G. anomala* and uninfected individuals. The clearest difference we observed was that infected fish were more social. Further, infected fish tended to be somewhat more active and bold. Parasite load did not explain observed behavioural variation. Taken together, this suggests that parasite infection could explain variation in personality, dependent on the personality gradient in focus, which enables speculations of potential underlying mechanisms that could result in differences in host behaviour via parasite infection.

Differences in personality related to parasite infection status can, in principle, be due to one of two scenarios: parasites can change host behaviour, or pre-existing personality differences can influence an individual’s likelihood of parasite infection. Whether the first scenario results from a side effect of infection or direct parasite-host manipulation could potentially be understood by investigating which personality traits are influenced by the infection. For example, if overall activity is reduced, or activity in a feeding situation is increased, it may be a side effect of infection explained by depleted host nutritional state. If instead the host’s behaviour is affected in a way that directly increases transmission of the parasite, this can point towards parasite-host manipulation. The affected behaviour should in this scenario be specific to the parasite in question and to its mode of transmission (Lélu et al. 2013). For example, a parasite with horizontal transmission would be predicted to increase interactions with conspecifics, while increased risk-taking would be predicted if the final host is a predator of the infected individual.

The differences in personality we observed in our fish may be the result of a host strategy to compensate for energetic depletion resulting from infection, or a direct manipulation of host personality by the parasite to increase the possibility of transmission (Tripet and Richner 1997; Ward et al. 2005; Barber et al. 2017). We observe some similarity in behavioural differences in our study to studies that compared infected and uninfected sticklebacks with the parasite *S. solidus* (boldness, activity, Giles 1983; Tierney et al. 1993; Barber et al. 2004; Grécias et al. 2017). This suggests a similar outcome of infection, across these common parasites. Influences on nutritional state are here likely, and match that size had some relation to some of our measured behaviour. Nutritional state can affect fish positioning within the shoal in healthy sticklebacks (Ward et al. 2004), like in other host species (*Melanotaenia duboulayi*, Hansen et al. 2016), or other behavioural differences in infected fish (Godin and Sproul 1988; Rahn et al. 2015). We did not observe a quantitative behavioural response matching parasite load (but see, Giles 1983; Godin and Sproul 1988). This may suggest that direct manipulation by the parasite is less likely. However, the parasite could manipulate the host’s behaviour, but in a way such that the change in behaviour is only apparent up to a certain threshold in the parasite load. These speculations need further investigations to be clarified.

Because we used naturally infected individuals, our findings may be because certain personality types were more prone to parasite infection (Cavigelli 2005; Beldomenico and Begon 2009; Barber and Dingemanse 2010; Barron et al. 2015). Differences in personality can affect an individual’s response to their environment such as movement and space use, which, in turn, can affect parasite load and transmission (Sih et al. 2018). In chipmunks (*Tamias minimus*), more exploratory individuals hosted a greater abundance of ectoparasites compared to less exploratory individuals (Bohn et al. 2017). Bolder, more active female firebugs (*Pyrrhocoris apterus*) were more infected by mites (*Hemipteroseius adleri*) than their conspecifics that behaved in a less explorative way (Gyuris et al. 2016). It is plausible that bolder, more explorative sticklebacks are at a higher risk of infection due to increased encounter with the *G. anomala* parasite. Similarly, more social fish that tend to aggregate in shoals have a higher probability of exposure to infection (Barber et al. 2017). Our understanding of the relationship between parasite and host could be improved by artificially induced infection of uninfected fish under laboratory conditions, to determine if the behavioural differences observed in infected fish are indeed caused by the parasite. A similar approach has been successfully used in the stickleback-*S. solidus* model confirming causality of observed relationships (Barber and Scharsack 2010) and in other species (crustacean hosts of microsporidian parasites, Rode et al. 2013; Eurasian minnows artificially infected with the trematode *Diplostomum phoxini*, Kekäläinen et al. 2014).

Speculations into the potential mechanisms by which *G. anomala* can influence hosts come from the stickleback-*S. solidus* system, in which work has shown altered monoamine neurotransmitter patterns in several brain areas of infected fish (Øverli et al. 2001). Confirming the role of the monoaminergic systems, in the crustacean *Gammarus pulex*, a serotonin injection mimicked parasite-host manipulation (Perrot-Minnot et al. 2014). Thus, monoaminergic systems could underlie variation in personality via parasite infection (e.g. Solbrig et al. 1996; Øverli et al. 2001; Adamo 2002; Biron et al. 2005; Shaw et al. 2009). Given the complex, digenetic life cycle of *S. solidus*, studies using artificial infection and the *G. anomala*-three-spined stickleback relationship may be a good model for the investigation of parasite infection and host personality and the mechanism by which parasite influences host.

Independent of the direction of causality in the link between personality and parasitism, the results we observed show different behaviour between infected and uninfected individuals. Thus, parasitism can play an important role in explaining observed variation in personality and thus in the evolutionary ecology of animal personality (Poulin 1994; Lefevre et al. 2009; Barber and Dingemanse 2010; Kortet et al. 2010; Thomas et al. 2010). This further suggests that individual personality traits can be linked to, and a consequence of, selection and adaptation resulting from parasite infection.

## Conclusions

Our study demonstrates that three-spined sticklebacks, naturally infected by the parasite *G. anomala*, display differences in personality compared to uninfected conspecifics. Specifically, we show that infected individuals are more sociable than uninfected fish. Parasitism could thus explain some of the observed variation in host personality. Future studies need to establish the causality in the link between parasite infection and host personality traits in the *G. anomala*-stickleback system, why only certain aspects of personality were affected by the parasite infection, and potential fitness benefits for the parasite.

## Electronic supplementary material

To calculate repeatability of behaviour, we used the ‘rpt’ function (package rptR; Stoffel et al. 2017).

ESM 1 (XLSX 22 kb)
